# Supplementary material for: Guaranteed efficient energy estimation of quantum many-body Hamiltonians using ShadowGrouping
Source: Nat Commun. 2025 Jan 15;16:689. doi: 10.1038/s41467-024-54859-x (PMC11735636; doi:10.1038/s41467-024-54859-x)
Supplement: Supplementary file 1 — Supplementary Information [file 41467_2024_54859_MOESM1_ESM.pdf]

Supplementary Information: Guaranteed efficient energy estimation of quantum many-body Hamiltonians using ShadowGrouping

Alexander Gresch[1, 2, *] and Martin Kliesch[2, †]

[1] *Faculty of Mathematics and Natural Sciences, Heinrich Heine University Düsseldorf, Germany*

[2] *Institute for Quantum Inspired and Quantum Optimization, Hamburg University of Technology, Germany*

In this Supplementary Information, we provide further details on the two main paradigms for the measurement reduction, namely grouping schemes and shadow methods in the first two sections, respectively. Afterwards, we prove our main results: first, the energy estimation tail bound in the third section, and afterwards, a rigorous criterion for when a Hamiltonian truncation is justified in the fourth one. Secondly, we follow up with the respective proof of the hardness result. We conclude with further numerical results to supplement the numerical benchmark within the respective result's subsection.

I. GROUPING METHODS

Grouping schemes make use of the fact that in Equation (2), many of the Pauli operators commute with each other which allows them to be measured simultaneously using the same measurement circuit. To this end, we want to decompose the operator collection $\{O^{(i)}\}$ into N_g possibly overlapping sets of commuting operators where N_g should be made as small as possible. This is called grouping of Pauli observables. This way, we only require $N_g \ll M$ independent measurement settings which reduces the total run-time of the sampling procedure. In principle, for a system of n qubits, the decomposition in Equation (2) can have up to $M_{\max} = 4^n - 1$ relevant terms each requiring individual read-out. In comparison, this collection can be partitioned into $N_g^{\min} = 2^n + 1$ equal groups of $2^n - 1$ members each, which roughly corresponds to a quadratic reduction in the number of circuit preparations. However, optimally grouping a given collection referred to as MIN-GROUPING, that is finding the partition with $N_g^{\min}$, is NP-hard in the system size [40]. As a trade-off between the estimation routine's run-time and this costly grouping step, approximate grouping algorithms have been devised, see the Introduction for details. Lastly, partitioning into commuting groups generally requires measurement circuits consisting of multiple two-qubit gates. As they increase the noise level of the quantum circuit significantly, we restrict ourselves to single-qubit gates only and, thus, constraining general commutativity to qubit-wise commutativity (QWC). Due to the tensor structure of the Pauli observables it is easy to see that QWC implies general commutativity. The contrary is not true: $\mathrm{C}[XX, YY] = \text{true}$ but $\mathrm{C}_{\mathrm{QWC}}[XX, YY] = \text{false}$. For grouping operators that fulfill QWC, polynomial heuristic algorithms of low degrees are applicable to efficiently find a solution which, however, may not be optimal [1].

II. SHADOW METHODS

The method of classical shadows is to find a classical approximation $\hat\rho$ to a quantum state ρ that reproduces the same expectation values for an ensemble of M observables $\{O^{(i)}\}\ \forall i \in [M]$ up to some error threshold ϵ [7, 8]. A classical shadow is constructed by rotating the target state ρ via a randomly drawn unitary $U \in \mathcal{U}$ from a fixed ensemble $\mathcal{U}$, e.g., local Pauli operators and tensors thereof or Clifford gates. In consequence, the state transforms as $\rho \mapsto U\rho U^\dagger$. Afterwards, the resulting state is measured in the computational basis which yields a bit string $\boldsymbol{b} \in \{0,1\}^n$ of n bits with probability $\langle \boldsymbol{b}\,|U\rho U^\dagger|\,\boldsymbol{b}\rangle$. Storing this measurement outcome is efficiently done on a classical computer and only scales linearly with the number of qubits n. The rotation is undone by applying the adjoint of the chosen unitary U onto $|\boldsymbol{b}\rangle$. This yields the state $U^\dagger|\boldsymbol{b}\rangle\langle\boldsymbol{b}|U$. This procedure can be repeated multiple times, e.g., as often as the measurement budget allows it. The expectation over all unitaries $U \in \mathcal{U}$ and the corresponding measurement outcomes $\boldsymbol{b}$ is a Hermitian map $\mathcal{M}$ for the underlying quantum state ρ:

$$\mathcal{M}(\rho) = \mathop{\mathbb{E}}_{U}\mathop{\mathbb{E}}_{\boldsymbol{b}}\left[U^\dagger|\boldsymbol{b}\rangle\langle\boldsymbol{b}|U\right] = \mathcal{M}^\dagger(\rho). \tag{1}$$

Although the result may not be a valid quantum state (it is not necessarily positive-semidefinite), $\mathcal{M}$ can still be regarded as a quantum channel and inverted. Since $\mathcal{M}$ is a linear function, we can recover the state ρ in expectation via

$$\rho = \mathop{\mathbb{E}}_{U}\mathop{\mathbb{E}}_{\boldsymbol{b}}\left[\mathcal{M}^{-1}\left(U^\dagger|\boldsymbol{b}\rangle\langle\boldsymbol{b}|U\right)\right] \tag{2}$$

as long as $\mathcal{M}$ is tomographically complete. The completeness ensures invertibility and is defined as $\forall \rho, \sigma$ with $\rho \neq \sigma\ \exists U \in \mathcal{U}, |\boldsymbol{b}\rangle :\ \langle \boldsymbol{b}\,|U\sigma U^\dagger|\,\boldsymbol{b}\rangle \neq \langle \boldsymbol{b}\,|U\rho U^\dagger|\,\boldsymbol{b}\rangle$. The right choice of the transformation ensemble $\mathcal{U}$ ensures that the channel inversion can be applied efficiently by a classical computer. Surely, averaging over all possible ensemble unitaries and their respective outcomes is not efficiently possible. However, we are not interested in a

* alexander.gresch@hhu.de

† martin.kliesch@tuhh.de

full description of ρ, but only in its expectation values. The latter is achievable by taking single snapshots called the classical shadows $\hat{\rho} = \mathcal{M}^{-1}(U^\dagger|\boldsymbol{b}\rangle\langle\boldsymbol{b}|U)$ and computing their empirical mean (12). This mean concentrates exponentially quickly around ρ (with $\mathbb{E}\hat{\rho} = \rho$) and the expectation value of any observable O is recovered in expectation:

$$o = \mathrm{Tr}[O\rho] = \mathrm{Tr}[O\mathbb{E}\hat{\rho}] = \mathbb{E}\,\mathrm{Tr}[O\hat{\rho}] = \mathbb{E}\hat{o}. \tag{3}$$

A. Locally-biased classical shadow

As stated above, classical shadows rely on sampling new measurement settings uniformly at random. The aim of locally-biased classical shadows (LBCSs) is to alter the underlying distribution β from which these settings are sampled in order to take the observables' structures, such as their mutual commutativity relations, into account [9, 11]. The sampling distribution β factorizes over the n qubits as

$$\beta(Q) = \prod_{i=1}^{n} \beta_i(Q_i)\,. \tag{4}$$

In the unbiased case above, we simply set $\beta_i = 1/3\ \forall i$, i.e., we assign the measurement basis uniformly at random and construct an unbiased estimator for the target observable O. By examining the variance of the estimator, we can bias the $\{\beta_i\}$ in such a way that the variances are decreased the most while retaining an unbiased estimator. Optimizing the sampling distribution is done in a preprocessing step. Additionally, it can readily be adapted to a weighted collection of target observables such as the ones in the Hamiltonian decomposition (2). Lastly, the sampling distribution can also be altered such that it takes into account a partially assigned measurement setting [11].

B. Derandomization

The idea of Derandomization is to greedily select the most advantageous measurement settings as indicated by the current inconfidence bound [10]. As we want to fulfill the ϵ-closeness of Equation (15) in the ∞-norm, we focus on each observable independently. We can regard each summand $\prod_{i,O_i\neq\mathbb{1}} \hat{b}_i \in \{-1,+1\}$ in Equation (12) as a random variable s with two possible outcomes. Thus, Equation (12) is only the empirical mean $\hat{s}$ for s. We can invoke Hoeffding's inequality in this case to end up with

$$\mathbb{P}\left[\left|\hat{o}^{(i)} - o^{(i)}\right| \geq \epsilon\right] \leq 2\exp\left(-\frac{\epsilon^2}{2}N_i\right), \tag{5}$$

which is - by construction - valid $\forall i$, $\forall \epsilon > 0$ and $\forall N_i \geq 0$. Using a union bound over $i \in [M]$, we conclude

$$\mathbb{P}\left[\|\hat{\boldsymbol{o}} - \boldsymbol{o}\|_{\ell_\infty} \geq \epsilon\right] \leq \sum_{i=1}^{M} 2\exp\left(-\frac{\epsilon^2}{2}N_i\right) \equiv \delta \tag{6}$$

from which a sample complexity (14) can be derived in the unweighted case. Moreover, Supplementary Equation (6) can be extended to cases where we have already partially assigned the next measurement setting [10]. This allows to update the inconfidence bound qubit by qubit. In this bound, the locality can still implicitly influence the values for N_i: Consider for example $O = X^{\otimes n}$. The probability of a random measurement setting Q to be compatible with O is exponentially small in the system size n: $\mathbb{P}[[O,Q] = 0] = 3^{-n}$. Thus, we require an exponential number of randomly drawn measurement settings compared to rightly choosing $Q = O$ instead. Randomly drawn classical shadows perform poorly as they completely disregard any structure in the target observables. The method of Derandomization aims to rectify this in a greedy approach: for each next allocation, the expected inconfidence bound is calculated and the Pauli operator is picked that bears the lowest bound. It has the advantage that it provably achieves a lower inconfidence bound than selecting the measurement basis uniformly at random. However, it enforces a fixed qubit ordering, and it is unclear how much different permutations affect the performance. In general, the disadvantage for any greedy algorithm is that optimal performance is not guaranteed and furthermore not probable.

III. PROOF OF THEOREM 3

In order to proof the theorem, we have to first take a step back and proof Theorem 6 and Corollary 7, subsequently. Afterwards, we make the connection to Theorem 3. However, we first need two helpful observations. The first theorem is proven in Ref. [45, Theorem 11] and Ref. [46, Chapter 6]. We use a slightly weaker instantiation of the variance bound for real-valued martingales:

Theorem 10: Let $X_1, \ldots, X_N$ be arbitrary B-valued random variables, s.t. $X_i \in L^2(B)$. Set $\mathbf{X}_i = \{X_1, \ldots, X_i\}$, $\mathbf{X}_0 = \varnothing$. Let $Z_0 = 0$ and let $Z_1, \ldots, Z_N$ be a sequence of real-valued random variables. Assume the martingale condition

$$\mathbb{E}[Z_i \mid \mathbf{X}_{i-1}] = Z_{i-1} \tag{7}$$

holds for $i = 1, \ldots, N$. Assume further that the martingale difference sequence $D_i = Z_i - Z_{i-1}$ respects

$$|D_i| \leq c_i\,, \quad |\mathbb{E}[D_i^2 \mid \mathbf{X}_{i-1}]| \leq \sigma_i^2\,. \tag{8}$$

Then, with $V = \sum_{i=1}^{N} \sigma_i^2$,

$$\mathbb{P}[Z_N > t] \leq \exp\left(-\frac{t^2}{4V}\right) \tag{9}$$

for any $t \leq 2V/(\max_i c_i)$.

In addition, we also make use of the following lemma, proven in Ref. [46, Lemma 6.16], see also Ref. [63]:

Lemma 11: Let $X_1, \dots, X_N$ be independent B-valued random variables. Let D_i as defined in Supplementary Equation (13). Then, almost surely for every $i \leq N$,

$$|D_i| \leq \|X_i\|_B + \mathbb{E}[\|X_i\|_B] \,. \tag{10}$$

Furthermore, if the X_i are in $L^2(B)$ we also have

$$\mathbb{E}[D_i^2 \mid \mathbf{X}_{i-1}] \leq \mathbb{E}[\|X_i\|_B^2] \,. \tag{11}$$

With this, we are ready for the proof.

Proof: We inherit its concentration inequality from the variance bound of martingales, Theorem 10. As our proof follows the one given in Ref. [45], we merely summarize the proof and highlight our contribution to the proof. The key idea is to approximate the zero-mean random variable $\|S\|_B - \mathbb{E}[\|S\|_B]$ (recall that $S := \sum_i X_i$.) by the martingale

$$Z_i = \mathbb{E}\left[\|S\|_B \mid \mathbf{X}_i\right] - \mathbb{E}[\|S\|_B] \,, \tag{12}$$

where $\mathbf{X}_i := \{X_1, \dots, X_i\}$. As suggested by Theorem 10, we define

$$D_i = Z_i - Z_{i-1} = \mathbb{E}\left[\|S\|_B \mid \mathbf{X}_i\right] - \mathbb{E}\left[\|S\|_B \mid \mathbf{X}_{i-1}\right] \,. \tag{13}$$

Then, Lemma 11 asserts that

$$\begin{aligned} |D_i| &\leq 2\max\|X_i\|_B =: c_i \\ \mathbb{E}[D_i^2 \mid \mathbf{X}_{i-1}] &\leq \mathbb{E}[\|X_i\|_B^2] =: \sigma_i^2 \,. \end{aligned} \tag{14}$$

Our contribution now consists of finding an upper bound to the expectation value $\mathbb{E}[\|S\|_B]$. Using first the triangle inequality and then Jensen's inequality for $\mathbb{E}[Z] \leq \sqrt{\mathbb{E}[Z^2]}$, we have that

$$\begin{aligned} \mathbb{E}[\|S\|_B] &\leq \sum_{i=1}^N \mathbb{E}[\|X_i\|_B] \leq \sum_{i=1}^N \sqrt{\mathbb{E}[\|X_i\|_B^2]} \\ &\equiv \sum_{i=1}^N \sigma_i =: \sqrt{V_B} \,. \end{aligned} \tag{15}$$

Finally, inserting Supplementary Equation (12) into Theorem 10 and shifting $\mathbb{E}[\|S\|_B]$ over yields Theorem 6.

Up to now, we have not restricted ourselves on any particular choice of Banach space B. However, for certain choices of Banach spaces, we can tighten the upper bound on $\mathbb{E}[\|S\|_B]$ further. This is encapsulated by Corollary 7, which we prove in the following.

Proof: Let $1 \leq p < \infty$ for now. We define the space $L^p(B)$ the space of all B-valued random variables X such that

$$\mathbb{E}\|X\|^p = \int \|X\|_B^p \, \mathrm{d}\mathbb{P} < \infty \,. \tag{16}$$

The spaces $L^p(B)$ are indeed Banach spaces again with the norm $\|\cdot\|_{L^p}$ defined as

$$\|X\|_{L^p} = \left(\mathbb{E}\left[\|X\|_B^p\right]\right)^{1/p} . \tag{17}$$

For $B = L^2(\mathbb{R}^d)$, the vector space of d-dimensional real vectors equipped with the standard 2-norm, we can invoke Pythagoras' theorem due to the independence. Together with the zero-mean property of the X_i (as assumed) we arrive at

$$\mathbb{E}[\|S\|_{\ell_2}^2] = \sum_{i=1}^N \mathbb{E}[\|X_i\|_{\ell_2}^2] = \sum_{i=1}^N \sigma_i^2 \equiv V \tag{18}$$

$$\Rightarrow \mathbb{E}[\|S\|_{\ell_2}] \leq \sqrt{\mathbb{E}[\|S\|_{\ell_2}^2]} = \sqrt{V} \leq \sqrt{V_B} \,, \tag{19}$$

using Jensen's inequality again and the sub-additivity of the square root in the last step. This improvement also holds for $B = L^p(\mathbb{R}^d)$ with $p \in [1, 2]$ as

$$\begin{aligned} \mathbb{E}[\|S\|_{\ell_p}] &= \|S\|_{L^p(\mathbb{R}^d)} \leq \|S\|_{L^2(\mathbb{R}^d)} \\ &= \sqrt{\mathbb{E}[\|S\|_{\ell_2}^2]} = \sqrt{V} \,, \end{aligned} \tag{20}$$

where the norm relation stems from Lyupanov's inequality [64, Theorem 3.11.6].

Finally, we have all the tools required to prove Theorem 3:

Proof: We will apply Corollary 7 to random vectors $X_k := \boldsymbol{v}_k$ in $\mathbb{R}^M$ whose construction we explain below. Setting $V = \sigma^2 \geq \sum_{k=1}^N \mathbb{E}\|\boldsymbol{v}_k\|_{\ell_p}^2$ and $b \geq \|\boldsymbol{v}_k\|_{\ell_p}$, Equation (17) asserts to

$$\mathbb{P}\left(\Big\|\sum_{k=1}^N \boldsymbol{v}_k\Big\|_{\ell_p} \geq \epsilon\right) \leq \exp\left(-\frac{1}{4}\left[\frac{\epsilon}{\sigma} - 1\right]^2\right), \tag{21}$$

where $\sigma \leq \epsilon \leq \sigma^2/b + \sigma$. We now construct the random vectors as follows. Assume a list of N measurement settings $\boldsymbol{Q} \in (\mathcal{P}^n)^N$ such that it contains $N_i(\boldsymbol{Q}) > 0$ compatible settings for each target observable $O^{(i)}$. This allows us to construct the k-th random vector $\boldsymbol{v}_k$ such that it contains a non-trivial entry for the i-th observable if it is compatible with the k-th measurement setting and zero otherwise. In a sense, we embed the vector of measurement results for each observable with which the measurement setting is compatible (w.r.t. the respective compatibility measure C) into a larger vector by appropiately adding a zero for all non-compatible observables. With this construction, we can still make use of correlated samples since entries of random vectors do not need to be independent of each other (which they are not, in general: the zero entries depend on the non-zero ones, both ultimately depend on the chosen measurement setting). The independence between random vectors, however, is kept as each of the N measurement rounds is independent of the others. Having this construction idea in mind, each entry of the random vectors consists of the difference of the sampled value from the actual (but unknown) expectation value of the target observable, weighted by the corresponding factor h_i from the decomposition (2). Furthermore, we down-weight its importance by N_i to turn the summation over the vectors into an empirical mean while not counting the extra zero entries. We obtain N

vectors $\boldsymbol{v}_k$ with M entries each. Let $\hat{o}_k^{(j)}$ denote the k-th sample for the j-th target observable $O^{(j)}$ and $o^{(j)}$ again its mean value. The definition, thus, reads as

$$\begin{aligned} \boldsymbol{v}_k &= \left(v_{k,j}\right)_{j=1}^{M} \\ \text{with } v_{k,j} &:= \frac{h_j}{N_j}\left(\hat{o}_k^{(j)} - o^{(j)}\right)\left[\mathrm{C}[O^{(j)}, Q_k]\right] \end{aligned} \tag{22}$$

Here, $[\,\cdot\,]$ denotes the Iverson bracket that asserts to 1 for a true argument and 0 else. The $\boldsymbol{v}_k$ has zero mean by construction. We bound its norm from above by the fact that $\mathrm{spec}(O^{(j)}) \subseteq [-1, 1]\ \forall j$ as we deal with tensor products of Pauli observables. The Iverson bracket can also be dropped because it can only decrease the actual value of the norm. We arrive at

$$\begin{aligned} \|\boldsymbol{v}_k\|_{\ell_1} &= \sum_{j=1}^{M} \frac{|h_j|}{N_j}\left|\hat{o}_k^{(j)} - o^{(j)}\right|\left[\mathrm{C}[O^{(j)}, Q_k]\right] \\ &\leq 2\sum_{j=1}^{M} \frac{|h_j|}{N_j} = 2\|\boldsymbol{h''}\|_{\ell_1} =: b \end{aligned} \tag{23}$$

with $h''_j = h_j/N_j$. A similar trick is done for the expectation value of its square, i.e.,

$$\begin{aligned} \mathbb{E}[\|\boldsymbol{v}_k\|_{\ell_1}^2] &\leq \mathbb{E}\left[\left(2\sum_{j=1}^{M} \frac{|h_j|}{N_j}\ \left[\mathrm{C}[O^{(j)}, Q_k]\right]\right)^2\right] \\ &= 4\left(\sum_{j=1}^{M} \frac{|h_j|}{N_j}\ \left[\mathrm{C}[O^{(j)}, Q_k]\right]\right)^2, \end{aligned} \tag{24}$$

in order to drop the expectation altogether. With this, we proceed by calculating a bound σ^2 on the expected sample variance as

$$\begin{aligned} &\sum_{k=1}^{N} \mathbb{E}\|\boldsymbol{v}_k\|_{\ell_1}^2 \\ &\leq 4\sum_{i,j=1}^{M} \frac{|h_i h_j|}{N_i N_j} \sum_{k=1}^{N}\left[\mathrm{C}[O^{(i)}, Q_k]\right]\left[\mathrm{C}[O^{(j)}, Q_k]\right] \\ &\leq 4\sum_{i,j=1}^{M} \frac{|h_i h_j|}{N_i N_j} \min(N_i, N_j) \\ &\leq 4\sum_{i,j=1}^{M} \frac{|h_i h_j|}{N_i N_j}\sqrt{N_i N_j} = 4\sum_{i,j=1}^{M} \frac{|h_i h_j|}{\sqrt{N_i N_j}} \\ &= 4\|\boldsymbol{h'}\|_{\ell_1}^2 =: \sigma^2, \end{aligned} \tag{25}$$

where we have first used Supplementary Equation (24), then summed over k by using the definition of $N_{i/j}$. The second inequality arises from the fact that $\min(a, b) \leq \sqrt{ab}$ for any non-negative real numbers a, b. Lastly, we have defined $(h')_j = h_j/\sqrt{N_j}$. The notation has been chosen such that each apostrophe to $\boldsymbol{h}$ indicates element-wise division by $(\sqrt{N_i})_i$.

For the sake of readability and clarity, we introduce

$$\mathrm{MEAN}(O)_{\boldsymbol{S}} := \frac{1}{|\boldsymbol{S}|}\sum_{i=1}^{|\boldsymbol{S}|} \hat{o}_i\,, \tag{26}$$

where the sum runs over the outcomes of the measurement settings $\boldsymbol{S}$ (with $S_i \in \boldsymbol{Q}$) that are compatible with O. This way, we reformulate the mean sample vector as

$$\begin{aligned} \sum_{k=1}^{N} \boldsymbol{v}_k &= \left(\sum_{k=1}^{N_j} \frac{h_j}{N_j}\left(\hat{o}_k^{(j)} - o^{(j)}\right)\right)_j \\ &= \left(h_j\left[\mathrm{MEAN}_{\boldsymbol{S}_j}(O^{(j)}) - o^{(j)}\right]\right)_j. \end{aligned} \tag{27}$$

This relates to the absolute energy estimation error as

$$\begin{aligned} |\hat{E} - E| &= \left|\sum_{j=1}^{M} h_j\left[\mathrm{MEAN}_{\boldsymbol{S}_j}(O^{(j)}) - o^{(j)}\right]\right| \\ &\leq \sum_{j=1}^{M}\left|h_j\left[\mathrm{MEAN}_{\boldsymbol{S}_j}(O^{(j)}) - o^{(j)}\right]\right| \\ &= \left\|\sum_k \boldsymbol{v}_k\right\|_{\ell_1}. \end{aligned} \tag{28}$$

The first inequality step is the general triangle inequality for real numbers and the last step follows by evaluating the 1-norm of Supplementary Equation (27). Putting Supplementary Equations (22), (23) and (25) into Corollary 7 and, furthermore, using Supplementary Equation (28), we have proven Equation (4).

Lastly, we deduce Equation (5). Let $\delta \in (0, 1/2)$. We set the right-hand side of the latter equal to the given δ. Solving for ϵ yields

$$\begin{aligned} \epsilon &= \alpha_\delta\|\boldsymbol{h'}\|_{\ell_1} \\ \alpha_\delta &:= 4\sqrt{\log(1/\delta)} + 2\,. \end{aligned} \tag{29}$$

Using the observation (the claim can be, e.g., derived by taking the derivative on both sides) that

$$\frac{3}{2}x^2 \geq x + \frac{1}{2} \quad \forall x \geq 1\,, \tag{30}$$

we have that $\alpha_\delta \leq 6\log(1/\delta)$, hence Equation (5).

IV. THE TRUNCATION CRITERION

The guarantee based on Theorem 3 quantifies the total statistical noise of measuring the respective Pauli terms in Equation (2). In order to decrease the measurement effort, a popular approach has been to truncate the Hamiltonian decomposition, i.e., to remove terms of small coefficient magnitude altogether [20]. This procedure introduces a systematic error which can be compared to the statistical error of the untruncated decomposition. Due to the form

of Equation (5), the statistical errors of the individual terms add up independently of each other. As a result, this allows us to compare the statistical error contribution of each observable with its systematic error upon truncation. In essence, given a list of measurement settings $\boldsymbol{Q}$, one has to update the empirical estimate $\hat{o}^{(i)}$ for each observable in the decomposition as

$$\hat{o}^{(i)} \leftarrow \begin{cases} 0 & \text{if } N_i < \alpha_\delta^2\,, \\ \hat{o}^{(i)} & \text{if } N_i \geq \alpha_\delta^2\,, \end{cases} \tag{31}$$

with α_δ in Supplementary Equation (29).

This truncation criterion (31) ensures the optimal trade-off between the statistical error in Equation (4) and the systematic one due to truncation. This is formalized in the following.

Corollary 12: Consider the setting of Theorem 3 and let $\delta \in (0, 1/2)$. Then, with probability at least $1-\delta$, the application of the truncation criterion (31) leads to a provably higher precision.

Importantly, the criterion does not depend on the magnitude of the coefficients $|h_i|$. We visualize it in Figure 1 to illustrate that a feasible number of compatible settings is required even for $\delta \ll 1$.

Proof: By virtue of Theorem 3, we compare the statistical error with the systematic error that is introduced by Supplementary Equation (31). Assume we leave out the i-th term in the grouped empirical mean estimator (3) by setting $\hat{o}^{(i)} = 0$. This introduces a symmetric systematic error of at most $\epsilon_{\mathrm{sys}}^{(i)} = |h_i|$, see Equation (13). Let I_{sys} be the index set of omitted terms and I_{stat} the index set of measured observables. Both sets are disjoint and their union yields the index set $[M]$. We write, using first the triangle inequality and then Supplementary Equation (29),

$$\begin{aligned} |E - \hat{E}| &\leq \sum_{i=1}^{M} \left| h_i \left(\hat{o}^{(i)} - o^{(i)} \right) \right| \\ &\leq \sum_{i \in I_{\mathrm{stat}}} \frac{\alpha_\delta}{\sqrt{N_i}} |h_i| + \sum_{i \in I_{\mathrm{sys}}} |h_i| \\ &:= \epsilon_{\mathrm{stat}} + \epsilon_{\mathrm{sys}} \end{aligned} \tag{32}$$

with probability $1-\delta$. This readily provides us with a criterion whether the error for the i-th term should be estimated by statistical or systematic means, i.e., whether it should be measured or simply set to zero: for each term in the decomposition, we simply inspect which summand of the sum yields the smaller value and include i. Comparing the corresponding entries in the two summations above, we arrive at Corollary 12.

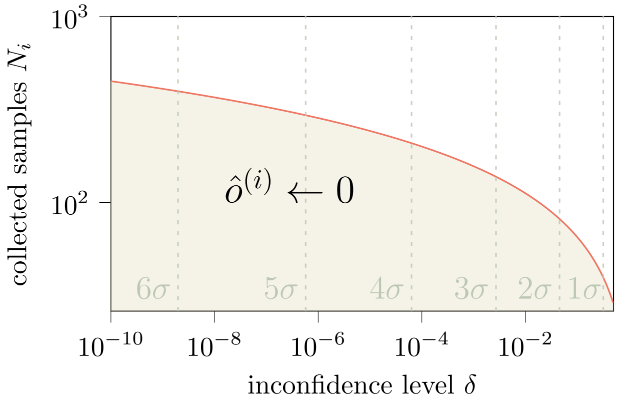

Supplementary Figure 1. Illustration of the truncation criterion (31). We plot α_δ^2 as a function of the selected inconfidence level δ (orange line). The criterion tells us that it is better to go with a truncation if $N_i \leq \alpha_\delta^2$ (shaded area). For illustrative purposes, we have added several σ-regions to the confidence levels. They suggest that reaching confidence levels that make significant deviations virtually impossible does not require an infeasible number of measurements per observable.

V. PROOF OF PROPOSITION 4

We prove the proposition by first stating a more general problem that translates the optimization over a finite set (here, the measurement settings) to the minimization of a target function. Subsequently, we show that this problem is NP-complete in the number of qubits n which even holds true for a single measurement setting. This is done by relating the problem to the one of MIN-GROUPING, i.e., grouping a given collection of Pauli observables into the smallest number of partitions with overlap which is NP-hard in the number of qubits [40] and to CLIQUE, i.e., finding the largest clique in a graph [47]. Proposition 4 then follows as a corollary.

To define the problem setting, we recall what makes a Pauli string compatible with a measurement setting. The concept of compatibility, Definition 2, is crucial for minimizing the right-hand side of Equation (4). We define the minimization problem as follows.

Problem 13: Fix a compatibility indicator, either $g = \mathrm{C}$ or $g = \mathrm{C}_{\mathrm{QWC}}$ and a family of convex and strictly monotonously decreasing functions $f_\alpha : \mathbb{R}_+ \to \mathbb{R}_+$ with $\lim_{x\to\infty} f_\alpha(x) = 0$, parametrized by $\alpha > 0$ such that $f_\alpha(0) > 0$ for all $\alpha > 0$ and $f_\alpha(x) < f_\beta(x)$ for all $x > 0$ and $\alpha < \beta$ which can be evaluated in polynomial time. Assume that α controls the curvature of f_α, i.e., $f_\alpha(1) = L_\alpha f_\alpha(0)$ with constant $L_\alpha < 1$ that can be made arbitrarily small by increasing either α or $1/\alpha$.

Input:

1. A set of weighted Pauli strings $\mathcal{O} = \{(O^{(i)}, h_i)\}_{i\in[M]}$ (comprising a Hamiltonian (2))
2. Measurement budget N

Output:

$$\mathcal{Q}_{\mathrm{opt}} := \operatorname*{arg\,min}_{\mathcal{Q} \in (\mathcal{P}^n)^N} \sum_{i=1}^{M} f_{|h_i|}(N_i(\mathcal{Q}))\,, \tag{33}$$

where $N_i(\mathcal{Q}) = \sum_{j=1}^{N} g(O^{(i)}, \mathcal{Q}_j)$. The corresponding decision version of the problem is to decide for a threshold

value $t \in \mathbb{R}$ as an additional input whether the optimal value is $\sum_{i=1}^{M} f_{|h_i|}(N_i(\mathcal{Q}_{\mathrm{opt}})) \leq t$.

The minimization of the right-hand side of Equation (4) is one of the instantiations of Problem 13. The function family f_α is derived in more detail around Equation (23) – in essence, we ensure that the right-hand side of Supplementary Equation (33) is nothing but $\|\boldsymbol{h'}\|_{\ell_1}$ from Equation (4). However, this problem class potentially includes other tail bounds as long as they decrease strictly monotonously with each of the N_i and are convex (see also Table I for another tail bound which is less tight). Now, we prove that BEST-ALLOCATION-SET is NP-hard in the number of qubits n. To this end, we find a polynomial time many-one reduction from the NP-hard MIN-GROUPING [40] to BEST-ALLOCATION-SET in the following.

Proposition 14: Problem 13 is
a) NP-complete.
b) NP-complete, even when restricted to $N = 1$.

Proof: The decision version of BEST-ALLOCATION-SET is in NP: given any $\mathcal{Q} \in (\mathcal{P}^n)^N$, we can efficiently calculate the argument of the right-hand side of Supplementary Equation (33) (because the $f_{|h_i|}$ can be evaluated in polynomial time) and compare it to the threshold value $t \in \mathbb{R}$.

BEST-ALLOCATION-SET is also NP-hard (Proposition 14a) since we can find a polynomial time many-one reduction from MIN-GROUPING: We are given a collection of Pauli observables $\{O^{(i)}\}_{i\in[M]}$ and a threshold value $\eta \in \mathbb{N}$ for the number of groups. We now construct the corresponding weights by inspecting the functions f_α. The key idea is to choose the weights in such a way that the minimization of the target function (33) requires each observable to have at least one compatible measurement. Let $\Delta_\alpha(x) = f_\alpha(x+1) - f_\alpha(x)$ be the slope of the secant between x and $x+1$. Because of convexity and monotony, we have that $\Delta_\alpha(0)/\Delta_\alpha(1) > 0$. Moreover, we have that $\Delta_\alpha(1) \leq f_\alpha(1) = L_\alpha$ as f_α is non-negative. Thus,

$$\frac{\Delta_\alpha(0)}{\Delta_\alpha(1)} \geq \frac{\Delta_\alpha(0)}{L_\alpha f_\alpha(1)} = \frac{1-L_\alpha}{L_\alpha} = \frac{1}{L_\alpha} - 1\,, \tag{34}$$

and there exists a constant γ such that $\Delta_\gamma(0) > M\Delta_\gamma(1)$. We use this constant to provide the set of weighted Pauli strings $\mathcal{O} = \{(O^{(i)}, \gamma)\}_{i\in[M]}$. Finally, set $N = \eta$. Let $\mathcal{Q}^* = \{Q_i \in \mathcal{P}^n\}_{i=1}^N$ be an optimal solution of BEST-ALLOCATION-SET. Dropping duplicates (with a worst-case time complexity of $\mathrm{O}(N^2)$) provides measurement settings $\mathcal{Q}_{\text{filt.}} \subset (\mathcal{P}^n)^k$ with $k \leq \eta$. Now we explain that this 'filtered version' of $\mathcal{Q}$ exactly contains the optimal solution of MIN-GROUPING. To see this, we go through each $Q \in \mathcal{Q}_{\text{filt.}}$ and through each $(O, w) \in \mathcal{O}$ and append O to the group belonging to Q if $g(Q, O) = \text{true}$. Due to the choice of γ, we can obtain YES-instances of MIN-GROUPING of threshold η from YES-instances of BEST-ALLOCATION-SET with threshold $Mf_\gamma(1)$.

Now, we fix $N = 1$ beforehand and show NP-hardness (Proposition 14b) by a reduction from CLIQUE with threshold $\nu \in \mathbb{N}$, the size of the clique [47]. Given a graph $G(V, E)$, we employ the polynomial time reduction of Ref. [40, Algorithm 2] in order to obtain $M = |V|$ n-qubit observables where $n = M(M-1)/2 - |E|$. We turn these observables into a Hamiltonian with the same γ as above. Then, BEST-ALLOCATION-SET given a threshold of $(M-\nu)f_\gamma(0) + \nu f_\gamma(1)$ delivers the solution to CLIQUE.

Finally, we show that Proposition 4 is just an instantiation of Problem 13. We formalize and prove this in the following.

Corollary 15: Consider a Hamiltonian (2), state ρ and an estimator $\hat{E}$ (3) of $E = \mathrm{Tr}[\rho H]$ and a measurement budget $N \geq 1$. Fix a compatibility indicator, either $g = \mathrm{C}$ or $g = \mathrm{C}_{\mathrm{QWC}}$. Choose an $\epsilon > 0$ and pick one of the tail bounds from Table I for $|\hat{E} - E| \geq \epsilon$. Then, finding the measurement settings $\mathcal{Q}_{\mathrm{opt}} \in (\mathcal{P}^n)^N$ that yields the smallest upper bound to $\mathbb{P}[|\hat{E} - E| \geq \epsilon]$ is
a) NP-complete in the number of qubits n,
b) NP-complete, even when $N = 1$.

Proof: We now check that we fulfill the requirements for Problem 13. To this end, we check that the minimization of either of the two tail bounds of Table I can be cast as Supplementary Equation (33). The energy estimation inconfidence bound (4) is minimized if and only if $\sum_i |h_i|/\sqrt{N_i(\mathcal{Q})}$ is minimized, see Equation (22). This implies to choose $f_\alpha(x) = \alpha/\sqrt{x}$ for $x \geq 1$, fulfilling the requirements. As noted around Equation (24), the case for $x = 0$ is ill-defined. Since $N_i(\mathcal{Q}) \in \{0, 1, \ldots, N\}$, we can set $f_\alpha(0) \coloneqq \alpha^2(1-1/\sqrt{2}) + \alpha$ and interpolate between 0 and 1 with a second-degree polynomial (with coefficients being polynomials of α such that f_α is differentiable at $x = 1$. One can check that $\Delta_\alpha(0) \geq \alpha\Delta_\alpha(1)$, i.e., it is easy to select $\gamma > M$ to finish the claim for this tail bound.

For the other tail bound, see e.g. Equation (29), we can readily select $f_\alpha(x) = \exp(-x/\alpha)$ which already fulfills all requirements from Problem 13. In this case, we have $\Delta_\alpha(0) \geq \exp(1/\alpha)\Delta_\alpha(1)$, i.e., selecting $\gamma < 1/\log(M)$ finishes the claim for the other tail bound.

VI. FURTHER BENCHMARK PLOTS

In Figure 3 (main text), we have shown the empirical benchmark, as captured by the RMSE (7), exemplarily for the NH_3-molecule for the Jordan-Wigner (JW) encoding. The same qualitative results also hold for the BK and the Parity mapping as well which we show in Supplementary Figure 2 for completeness' sake. We also append the results from the respective fitting routines for all considered molecules from the benchmark along with their extrapolation to reach $\epsilon_{\text{acc.}}^{\text{chem.}} = 1.6$ mHa. As fits, we again resorted to Equation (8) with respective uncertainty propagation from Equation (9). We again find ShadowGrouping improving upon the other methods when considering the largest problem instances (as

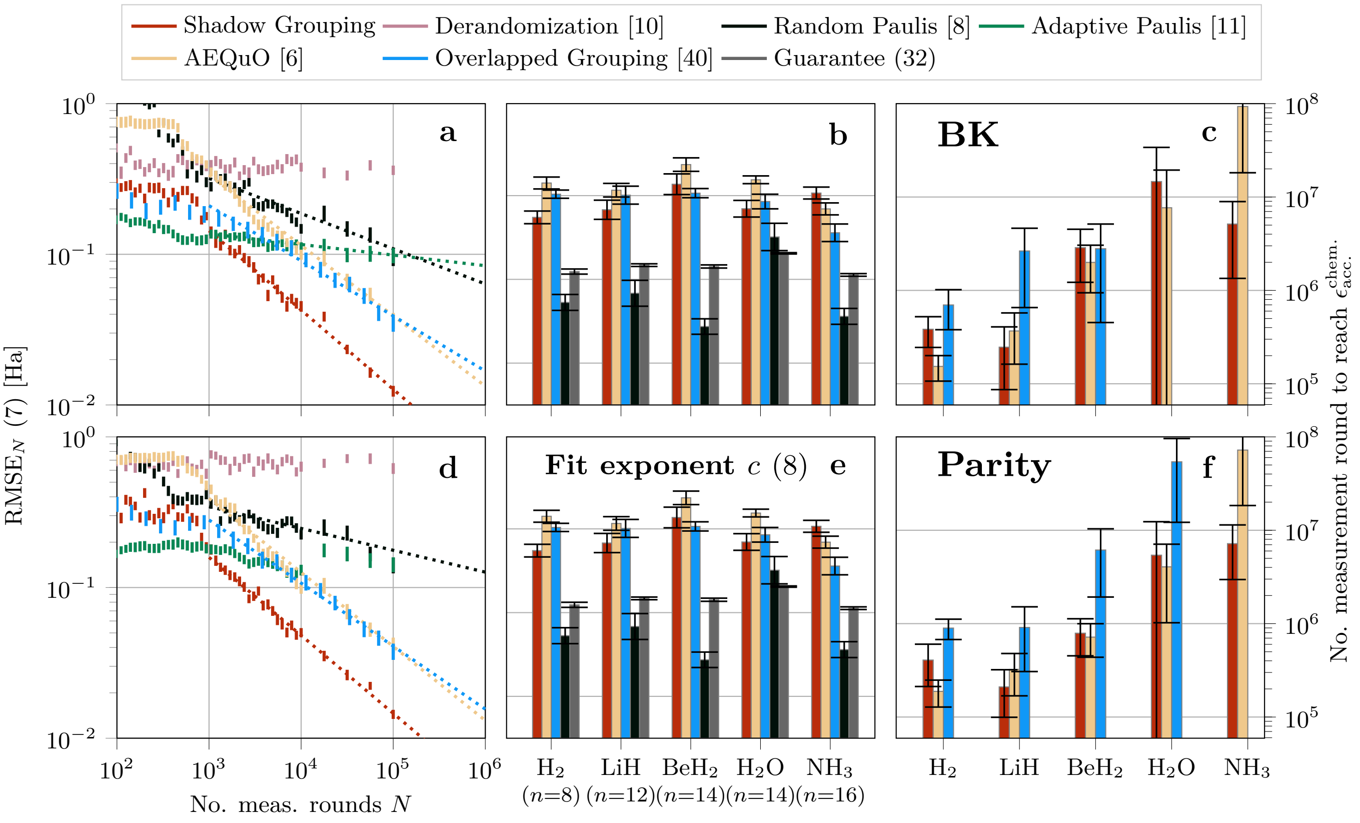

Supplementary Figure 2. Complementary plots to Figure 3 for the NH_3 molecule. a and d: empirical accuracy, b and e: their fit results, and c and f: extrapolations to $\epsilon_{\text{acc.}}^{\text{chem.}} = 1.6$ mHa. Error bars indicate the standard deviation of the RMSE, the fit uncertainties and propagated ones (9), respectively. a-c: results for the BK-encoded molecules; d-f: corresponding results for the Parity encoding. The most competitive empirical results typically reach a value for c of around 0.5. However, a look at the scaling of the corresponding guarantee (see Supplementary Figure 4) yields a lower value of around 1/3.

quantified by the system size n). Finally, we also add the fit parameter c obtained for our tail-bound based guarantee (32) as contrast to the empirical fit results. We comment on this in the next section in more detail.

For completeness' sake, we also provide results for states beyond the ground state (see Methods' subsection around Figure 5) for the BK and the Parity mapping in Supplementary Figure 3.

VII. COMPARISON OF GUARANTEES

In addition to the empirical analysis in the Results' section and the previous section of this Supplementary Information, Theorem 3 provides an upper bound to the actual accuracy for the energy estimation task given a measurement scheme that has generated a certain number of measurement settings N. We combine this with the truncation criterion of Corollary 12 to yield the smallest upper bound to $|\hat{E}-E|$, cf. Supplementary Equation (32). We employ the same measurement schemes from the literature as done in the corresponding Results' subsection and show their respective guarantees in Supplementary Figure 4. In the top row, we present the results akin to Figure 3, i.e., we plot the (averaged) accuracies as a function of the number of generated measurement settings N. Again, these plots follow a power law (8) with fit exponents c shown in the bottom row. Here, ShadowGrouping yields the highest exponents of a value around 1/3 which still is significantly below the ones of the empirical benchmark where $c \approx 0.5$. We attribute this discrepancy to the fact that not all terms commute pairwise with each other. Because Supplementary Equation (32) resembles a weighted power-mean w.r.t. the corresponding number of compatible measurement settings [55], this effectively decreases the resulting exponent c. In fact, if all terms were to commute pairwise, an exponent of $c = 1/2$ is recovered, see the discussion around Equation (33) in the corresponding Methods' subsection. Nevertheless, given these smaller fit parameters, we again extrapolate when the guarantee falls below chemical accuracy and provide the corresponding $N_{\text{acc.}}^{\text{chem.}}$ in the middle row. Here, the accuracy of ShadowGrouping is rigorously guaranteed (e.g., without assumptions on variance estimates) and is the best one known today for the applied measurement schemes. However, the values attained for $N_{\text{acc.}}^{\text{chem.}}$ are still far beyond feasibility, calling for a refinement of the tail bound in future works.

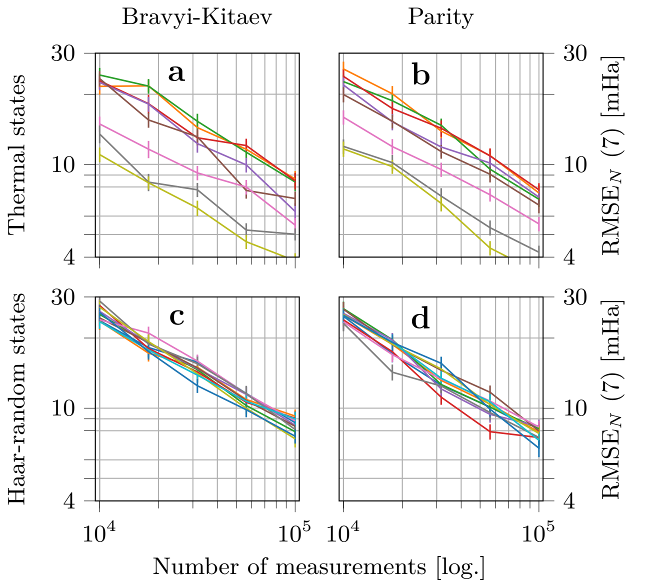

Supplementary Figure 3. Complementary plots to Figure 5. We subdivide the plot column-wise w.r.t. the BK and the Parity encoding and row-wise w.r.t. to the accuracy of estimating the energy of either the thermal state (34) (a and b) or a depolarized Haar-random one (35) (c and d).

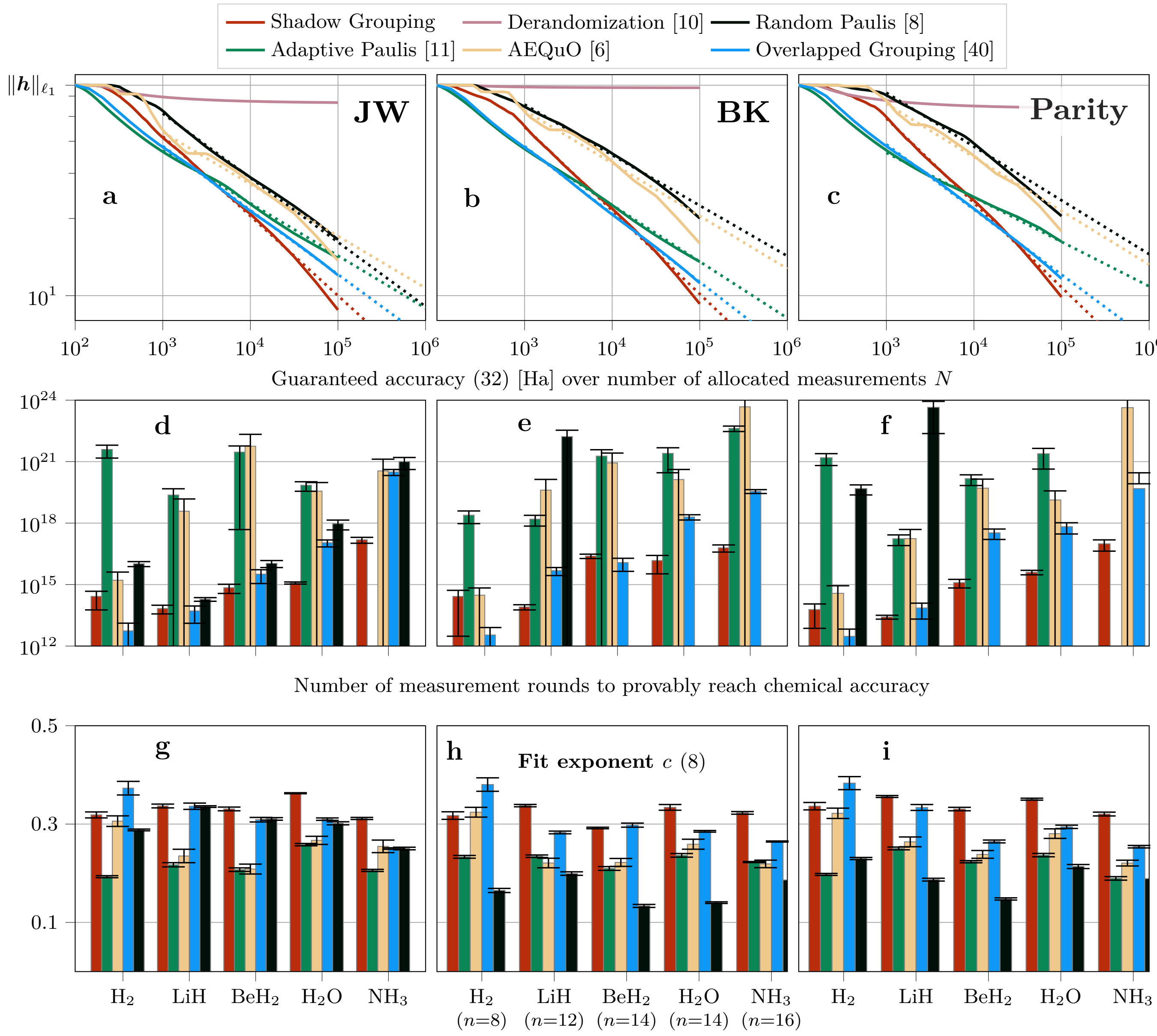

Supplementary Figure 4. Comparison of guarantees, Theorem 3, for the energy estimation task of the electronic structure problem for NH_3. a-c: Supplementary Equation (29) as a function of the number of generated measurements N, analogous to Figure 3 for the three fermion-to-qubit mappings. We have chosen $1-\delta = 98\%$ as confidence and report the upper bounds over a hundred independent runs in units of Ha. By construction, the guaranteed estimation accuracy is upper bounded by $\|\boldsymbol{h}\|_{\ell_1}$. d-f: The guarantees follow a power law (8) which allows to extrapolate to $N_{\mathrm{acc.}}^{\mathrm{chem.}}$, i.e., to reach an accuracy below 1.6 mHa, for the various molecules. g-i: The corresponding fit exponents are shown in the bottom row. Error bars indicate fit uncertainties and propagated ones (9). The various methods of the benchmark are discussed around Figure 3.
